# Content discovery and retrieval services at the European Nucleotide Archive

**DOI:** 10.1093/nar/gku1129

**Published:** 2014-11-17

**Authors:** Nicole Silvester, Blaise Alako, Clara Amid, Ana Cerdeño-Tárraga, Iain Cleland, Richard Gibson, Neil Goodgame, Petra ten Hoopen, Simon Kay, Rasko Leinonen, Weizhong Li, Xin Liu, Rodrigo Lopez, Nima Pakseresht, Swapna Pallreddy, Sheila Plaister, Rajesh Radhakrishnan, Marc Rossello, Alexander Senf, Dmitriy Smirnov, Ana Luisa Toribio, Daniel Vaughan, Vadim Zalunin, Guy Cochrane

**Affiliations:** European Molecular Biology Laboratory, European Bioinformatics Institute, Wellcome Trust Genome Campus, Hinxton, Cambridge CB10 1SD, UK

## Abstract

The European Nucleotide Archive (ENA; http://www.ebi.ac.uk/ena) is Europe's primary resource for nucleotide sequence information. With the growing volume and diversity of public sequencing data comes the need for increased sophistication in data organisation, presentation and search services so as to maximise its discoverability and usability. In response to this, ENA has been introducing and improving checklists for use during submission and expanding its search facilities to provide targeted search results. Here, we give a brief update on ENA content and some major developments undertaken in data submission services during 2014. We then describe in more detail the services we offer for data discovery and retrieval.

## INTRODUCTION

For 30 years, the European Nucleotide Archive (ENA) has been capturing, organizing and presenting openly the world's public domain sequencing data. ENA covers a spectrum of sequence data types, including raw reads, assemblies, functional annotation and contextual information, such as sample data and links into the scientific literature. Capturing data from all platforms, across all taxa and for all applications, the data resource continues to provide an essential foundation for the molecular life sciences.

Globally comprehensive data coverage, the fullest utility of our resource and the broadest reach to the scientific community are achieved through the collaboration of ENA with partner operations at the National Center for Biotechnology Information and the DNA Databank of Japan as part of the International Sequence Database Collaboration (INSDC; http://www.insdc.org) (1). In addition to the routine exchange of content, the collaboration provides the forum for the development and adoption of data standards and the focal point for interaction with scientific publishers.

A focus of our work in 2014 has been in the presentation of ENA content. As ENA content grows and its diversity increases, the need for rich user services supporting discovery, such as text and sequence search functions, and retrieval, such as web views of records and download functions, becomes greater. With developments both in web and programmatic interfaces, we have extended and enhanced our search services and have undergone a major revision of our website.

In this paper, we comment on growth in content over the year. We then highlight a selection of major developments in 2014 and detail our services for the discovery and retrieval of data.

## CONTENT AND GROWTH

ENA has continued to grow during 2014, reaching 1.2×10^15^ bases comprising almost 500 million assembled sequences and 9.5 trillion raw reads across more than one million taxa. With an overall footprint in excess of 2.5 petabytes, ENA data grow with a doubling time of approximately 20 months (see http://www.ebi.ac.uk/ena/about/statistics for live statistics). Notable data sets submitted to ENA during 2014 include the high-coverage genome sequence of a Neandertal individual from Denisova cave in the Altai (PRJEB1265; http://www.ebi.ac.uk/ena/data/view/PRJEB1265) (2) and Tara Oceans samples metabarcoding and shotgun sequencing of viruses, giruses, bacteria and protists (PRJEB402; http://oceans.taraexpeditions.org). Further interesting projects in the pipeline include metabarcoding and shotgun sequencing of epipelagic marine microbial samples from Ocean Sampling Day (PRJEB5129; http://www.microb3.eu/osd) and the genomic sequencing of coral, algal symbionts and associated microbial and viral communities from the Great Barrier Reef and Red Sea (PRJEB5277; http://www.barrierreef.org/our-research/research-we-support/solutions/sea-quence).

## MAJOR SERVICE DEVELOPMENTS

### Submission checklists

During 2014, ENA has expanded the annotation checklist system for submission of assembled and annotated sequences, releasing seven new checklists (Table 1). This brings the number of annotation checklists to 33 at the time of writing, with the complete list available at http://www.ebi.ac.uk/ena/submit/annotation-checklists. To improve users’ selection of checklists, and reduce erroneous use, all checklists have also been re-grouped within Webin (https://www.ebi.ac.uk/embl/genomes/submission/app/login/login.jsf). For sequence types where no suitable annotation checklist is currently available, submitters have the option to upload flat files prepared externally using the ‘Entry Upload’ option in Webin. It is possible to upload a full flat file or just feature table and sequence sections. In order to prepare flat files ready for upload, submitters can either use third party tools, which allow export of the annotation in the ENA supported flat-file format, or create their own flat files (http://www.ebi.ac.uk/ena/submit/sequence-submission). To facilitate the latter, ENA presents a number of flat-file examples for various sequence types that can be easily used as templates to structure annotation being submitted (http://www.ebi.ac.uk/ena/submit/entry-upload-templates). These templates provide the basic and minimal information required for the construction of a flat file. Advanced users who may wish to add further annotation within the scope of the INSDC feature table (1) are encouraged to refer to ENA WebFeat documentation (http://www.ebi.ac.uk/ena/WebFeat/).

**Table 1.** A list of seven recently released annotation checklists for submission of assembled and annotated sequences

Name	Description
Multi-exon gene	This checklist captures a complete or partial multi-exonic locus without needing to define splice sites or coding-region information. No translation will be generated.
ncRNA	For non-coding RNA (ncRNA) transcripts or single-exon genes of prokaryotic or eukaryotic origin with the exception of the ribosomal RNA (rRNA) and transfer RNA (tRNA).
Mobile element	This checklist captures a single complete or partial mobile element feature but does not allow for granular annotation of component parts, such as coding regions, repeat regions and miscellaneous features within the mobile element itself.
Multi-locus marker	This checklist captures **multiple** loci without the need to define the individual locus types beyond their names. Such examples are usually amplified regions containing tRNAs, rRNAs and coding genes from organelles, and mostly used for phylogenetic inference.
Viral untranslated region (UTR)	For complete or partial untranslated region (UTR) or nontranslated region (NTR) found at the termini of viral genomes.
Alphasatellite sub-viral particle	For submission of circular single-stranded DNA alphasatellite sequences associated with Begomovirus, Babuvirus and Nanovirus.
Plant viroid	For complete circular ssRNA plant viroid sequences.

For a complete list of all (33) currently available annotation checklists please refer to http://www.ebi.ac.uk/ena/submit/annotation-checklists.

Three new checklists have been added for use in capturing sample-related information: two supporting marine data samples (Micro B3 and Tara Oceans) and one for reporting metadata of pathogen samples for the Global Microbial Identifier reporting system (Global Microbial Identifier reporting standard checklist; GMI_MDM:1.1). Several existing sample checklists have also been updated to offer refined reporting standards and validation and more updates are currently in progress (e.g. GMI_MDM:1.1).

### Assembly submission

**Figure 1.**
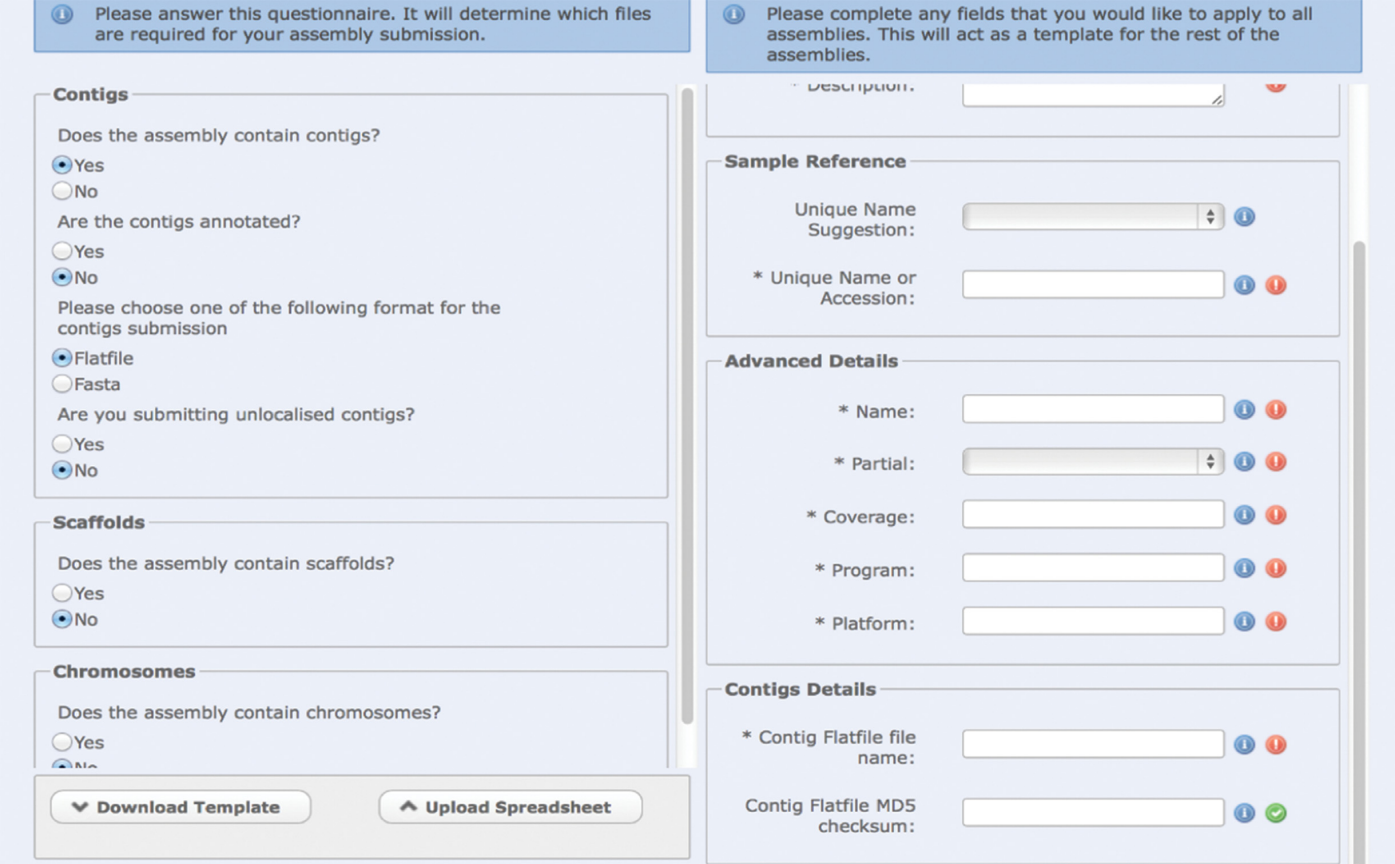
The Webin interactive decision tree available to assist users in submitting genome assemblies.

A new genome assembly submission service was deployed in early 2014, involving a major update to both the interactive and programmatic interfaces provided in our Webin system. This new functionality was developed to overcome three major challenges related to assembly submission: high complexity (many types of assembly spanning different data types); growing submission rates; and an insufficiently flexible internal data model (3). Prior to the new service, all genome assembly data required manual processing at ENA, resulting in a 40-day delay between submission and processing start time. In contrast, the new assembly submission service is an automated system and facilitates both single and multiple assembly submissions in single transactions. It provides flexibility regarding the data formats used (e.g. with support for FASTA, AGP, flat file) and minimum gap length reporting is supported as an alternative to full gap feature annotation. The internal assembly data processing pipeline has been parallelised and optimised against available compute capacity to enhance performance and throughput. Within Webin's interactive interface, the user is guided through a decision tree that prompts for provision of the necessary data files and descriptions of the assembly/assemblies (see Figure 1). For programmatic Webin users, the decision tree is provided within our documentation at http://www.ebi.ac.uk/ena/submit/programmatic-genome-submission.

Under the new system, 50% of all assembly submissions complete processing within 48 h of submission. Most of the remaining 50% of submissions have content-related issues requiring input from the submitter. While the model for this is for the submitter to rework the submission using Webin, ENA provides helpdesk support and in most cases these submissions are completed within 2 weeks of submission.

### Other developments

Another significant change to the Webin submission system in 2014 involved the unification of the two separate submitter account systems (for assembled/annotated sequences and for read data) and the development of a new authentication system. Previously, users needed to hold two separate accounts if their submissions spanned these different types of data. Old accounts have been merged as appropriate, so that all submitters now have a single account. The new authentication system allows more flexible log in options in interactive and programmatic Webin interfaces.

There has been a partial integration of the European Bioinformatics Institute's (EBI) BioSample database into ENA (4). New samples are assigned both an ENA and BioSample accession at time of submission with the BioSample accession made available within the sample XML, ENA browser and search services. Analysis, experiment and assembly submission all support the use of BioSample accessions. If a sample record does not yet exist within ENA, we obtain a copy from the BioSample database to link to the data. We are working on the introduction of update functionality for such samples.

In collaboration with INSDC partners and reflecting advances in sequencing technology, ENA has updated its read domain metadata schema to support new terms (relating to platform names, instrument models, library strategies and library selection methods). These and other changes as part of version 1.5 are detailed at http://www.ebi.ac.uk/ena/submit/read-xml-format-1--5#xml_changes_11_august_2014. In addition, we have altered the INSDC processes for inserting new terms to provide greater speed and flexibility in future.

ENA continues to host and contribute to training events including workshops, roadshows, webinars and online videos (http://www.ebi.ac.uk/ena/support). Feedback from these training events, in combination with user interaction via our helpdesk (datasubs@ebi.ac.uk), is used to shape our services.

## DATA DISCOVERABILITY AND RETRIEVAL

### Searching ENA data

There are three ways of searching content in the ENA browser and through programmatic services: Sequence (similarity) search, Text search and Advanced search. A search box within the header of all ENA web pages provides access to all three services for web users and programmatic access to these services is described fully at http://www.ebi.ac.uk/ena/browse/programmatic-access.

*Text search* allows the user's keywords to be located almost anywhere within ENA content, providing the simplest starting point for the exploration of the resource. However, while rapid and requiring no prior assumptions about usage of fields within ENA records, text searches can include false positives. For example, a user interested in fetching human sequences using the search term ‘human’ will also retrieve results for human-immunodeficiency virus, among other human viruses, and could pick up bacterial strains that have been isolated from the human gut. In late 2012, ENA introduced ‘Advanced search’ as a complementary service to specifically address these shortfalls, with the aim of enhancing data discoverability.

Sequences within ENA and the Ensembl and Ensembl Genomes reference assemblies that are similar to a sequence of interest can be found using *Sequence search*. While many users access this from the ENA home page, there are advanced options available from http://www.ebi.ac.uk/ena/search that offer control over the granularity of the search, such as by limiting to a taxonomic division or an Ensembl species, as well as the model for the search, that includes spliced, unspliced, translated and untranslated sequences. The results from a sequence similarity search include the percentage identity and E-value, as well as the ability to view alignments.

For these three search services, ENA content is organised into 11 domains (Table 2). All of these are available within *Advanced search*, all but ‘marker’ in *Text search* and ‘sequence’ and ‘non-coding’ alone in *Sequence search*.

**Table 2.** ENA data domains

Domain	Description
Assembly	Information describing the construction of reads and sequence contigs into higher order scaffolds and chromosomes
Sequence	Assembled and (optionally) annotated assembled reads
Coding	Sequence regions reported by data providers as being protein-coding regions
Non-coding	Sequence regions reported by data providers as representing non-protein-coding (RNA) genes
Marker	Information relating to phylogenetic, identification and molecular ecology marker data
Analysis	Derived data forms, such as recalibrated aligned reads, OTU tables and metabarcoding identifications
Read	Raw sequencing data from next generation platforms
Trace	Raw sequencing data from capillary platforms
Taxon	Information relating to the organism that was the source of the sequenced biological sample
Sample	Information relating to the biological sample studied in the sequencing experiment
Study	Information relating to the scope of the sequencing effort; also known as ‘Project’, the primary use of study is to unite content otherwise dispersed across the ENA domains

### Advanced search

ENA *Advanced search* was developed to provide a selectively searchable subset of contextual and functional annotation that leads users more directly to the content in which they are interested. Not only does the system make the fields harbouring these annotations searchable, but it also provides options for the user to configure views of the data with selected fields according to need. Since its inception, *Advanced search* has been expanding to include new domains with growing numbers of searchable fields, often in response to user or community requests, and further output formats such as a table-based report for use in down-stream workflows. At the time of writing, the service comprises 136 searchable and 157 returnable fields across 11 domains and 16 results, covering controlled vocabularies, dates, numbers, text, taxonomic classification and geospatial information. For a complete listing of these, please refer to http://www.ebi.ac.uk/ena/data/warehouse/usage.

The service is based upon a query language built on logical AND, OR and NOT, with defined taxonomic and geospatial functions in addition to standard text and numerical handling, giving the user finely grained control over a search. This query language is fully supported in the programmatic interface (http://www.ebi.ac.uk/ena/browse/search-rest) as well as via the ‘Edit query’ option of the web-based query builder (http://www.ebi.ac.uk/ena/data/warehouse/search). The taxonomic functions look at the taxonomic classification of the data rather than the presence of an organism name and also allow a sub-tree search to find, for example, not just sequences assigned the taxon *Mus musculus*, but also sequences for all sub-species and strains.

**Figure 2.**
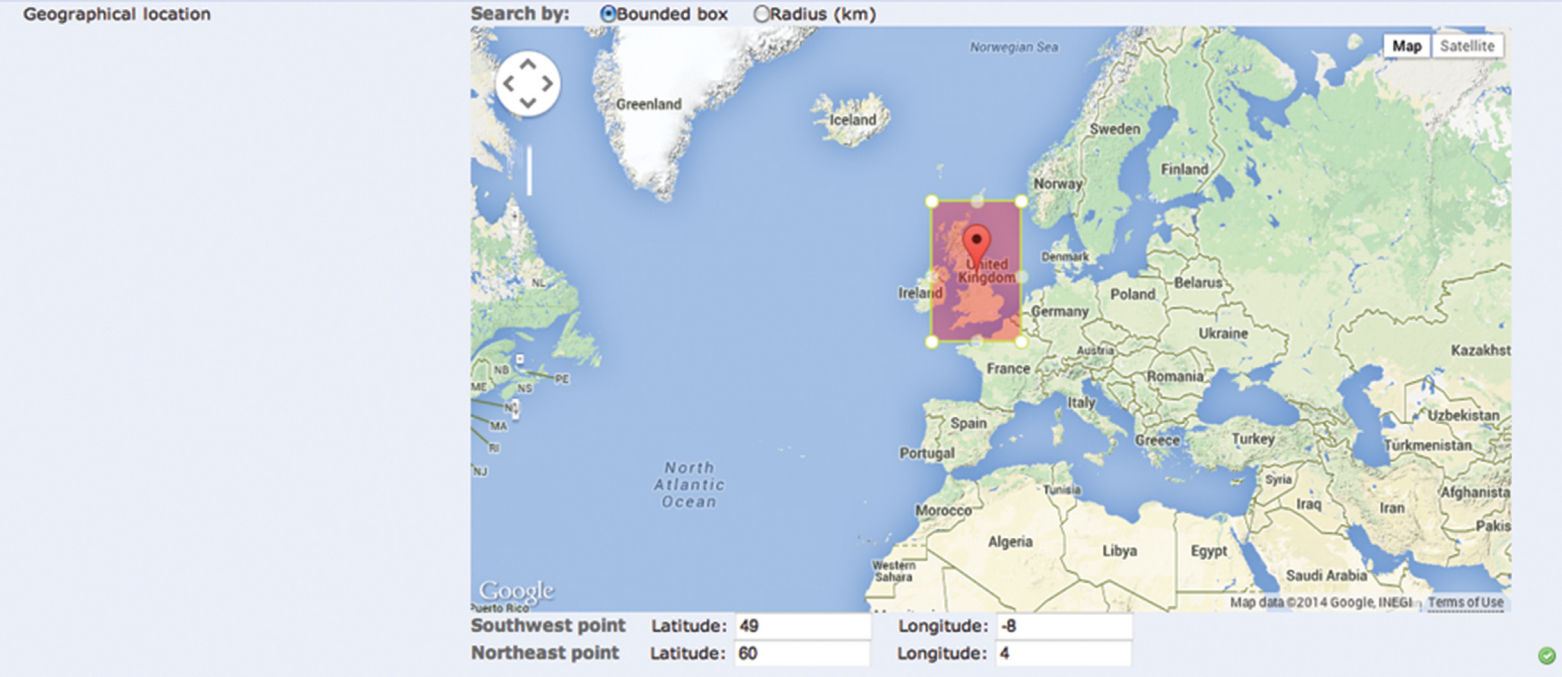
Reduced but simplified functionality for geospatial searching within the query builder.

With an increasing importance of georeferenced data (currently 7.3% and 4.1% of all public samples and sequences, respectively), there is a growing requirement to be able to locate data collected within a specific region. There are nine functions available providing different geometries for geospatial searching, allowing users to find samples collected at a given location, within a defined area, or within a certain radius of a point. Currently only two of these are available within the browser's query builder, but while full use of the geospatial functions via the REST URLs require knowledge of specific latitude and longitude ranges, the interactive option allows users to find the area of interest on a map (Figure 2).

*Advanced search* response pages present data of relevance to the search expression used. Under the default view, domains are summarised and, in cases, further broken down into ‘results’. Users can browse domains and results to see listings of records with thumbnail descriptions and onward links to the records themselves. The ‘Report’ tab provides an alternative to the list view that offers key contextual and functional annotations in tabular form, allowing the user to configure dynamically an appropriate selection of fields in the report. Options to download data are presented at appropriate points, offering a means to import and work further on data sets in external tools.

### Data processing for advanced search

One of the bigger challenges in providing an effective search of metadata and annotation remains the heterogeneity of the information submitted. While assembled and annotated sequences have a set of accepted structured qualifiers, there has traditionally been less formality in the attributes used to describe sample records associated with read data. ENA has developed a checklist-based system for sample records in which a set of attributes appropriate for a given study are defined for users and given mandatory or optional status according to importance. Values for these attributes are solicited during submission and used in the validation of incoming data. Many of these checklists—arguably the most mature of them—have been developed in collaboration with members of expert communities, such as the Minimal Information about any (x) Sequence (MIxS) checklists, with focus on environmental microbes (5), the Micro B3 checklists for marine data (http://www.ebi.ac.uk/ena/submit/microb3-checklist) and the epigenomics checklist for data from such methods as sequencing of chromatin immunoprecipitates (ChIP-Seq) (http://www.ebi.ac.uk/ena/submit/epigenomics-submissions). While the checklists provide defined sample attributes and formats for the study types that they cover, they do not as yet cover all classes of submitted study and it will be some time before thorough coverage is reached. In addition, because it is desirable to allow the reporting of extended information relevant for a specific study beyond those attributes defined in a checklist, the capacity for users to define their own attributes is retained. Given this system, it is difficult to prevent the submission of an optional attribute with a non-standard name and unconstrained value syntax. Examples of the scale of this problem are (not including case differences) the 28 currently known sample attributes used to describe latitude and longitude and the 17 different attributes that have been used to define collection date. Beyond the name of an attribute, a further issue is the syntactic form of its value. At last assessment, 22 such forms for latitude and longitude and 27 for collection date have been used.

A further challenge relates to differing scales and unit systems for numerical information. While in some cases, simple unit transformations into a single representation simply need some initial investigation (such as the standardisation of distances measured in feet and in metres), others are more challenging, for example, where ‘age’ can cover seconds to centuries. In some instances, a conversion is not possible, such as in the case where salinity is reported variously as a conductance (with unit μS cm^−1^) and as a concentration (with unit ppm, for example). Textual attributes can also prove to be problematic, particularly where there are conceptually few terms that describe the attribute. An example of this is seen in the text field ‘sex’ where, at last count, there were 74 different representations of ‘female’ (Table 3).

**Table 3.** How many ways can you say ‘female’? A list of values submitted in the ‘sex’ attribute for sequences and samples that all represent female

Different representations of ‘female’ submitted in sex qualifier/attribute		
18-day pregnant females	female (phenotype)	hexaploid female
2-yr-old female	female (pregnant)	individual female
3 female	female (worker)	lgb*cc females
400-yr-old female	female child	mare
adult female	female mice	metafemale
asexual female	female parent	monosex female
castrate female	female plant	normal female
cf.female	female with eggs	ovigerous female
cystocarpic female	female worker	oviparous sexual females
dikaryon	female, 6–8 weeks old	pseudohermaprhoditic female
dioecious female	female, other	remale
diploid female	female, pooled	semi-engorged female
f	female, spayed	sex: female
famale	female, virgin	sexual oviparous female
femail	female, worker	sf
femal	female(gynoecious)	sterile female
female	femalen	sterile female worker
female - worker	females	strictly female
female (alate sexual)	females only	tetraploid female
female (calf)	femele	thelytoky
female (f-o)	femlale	vitellogenic replete female
female (gynoecious)	gynoecious	worker
female (lactating)	healthy female	worker bee
female (outbred)	hen	worker caste (female)

Probably female (based on morphology).

Female (note: this sample was originally provided as a \‘male\’ sample to us and therefore labeled this way in the Brawand *et al*. paper and original geo submission; however, detailed data analyses carried out in the meantime clearly show that this sample stems from a female individual).

Accepting that heterogeneity will, at least for some substantial time, remain in the system, ENA maintains a set of rules that are used to map attributes to an Advanced search field and, for some fields, convert values into a consistent searchable format. The selection of treated attributes, formats, scales and values are scanned periodically, via manual curation, to update these rules, in an attempt to make the majority of data searchable. The ‘cleaned’ data are then made available for search and return in *Advanced search*, while the originally submitted information (and format) is retained. While this introduces some inconsistencies between raw downloadable data (presented as submitted) and search query and reports (‘cleaned’ version), this configuration respects the editorial control of the data submitter and mitigates degradation in cases where applied rules introduce inaccuracies.

In addition to these attribute-level rules, there are also rules applied across multiple attributes/qualifiers to group-related data together. This is in operation for phylogenetic marker sequences, where the domain ‘marker’ is constructed from relevant subsequences and annotation derived from the ‘coding’ and ‘non-coding’ domains (http://www.ebi.ac.uk/ena/data/warehouse/search?portal=marker). Information provided in the *gene*, *gene_synonym* and *product* qualifiers is used to define whether the sequence in question is a phylogenetic marker and, if so, which marker. An example of these rules, for Cytochrome C oxidase, subunit 1, can be found in Table 4.

**Table 4.** Rules applied during indexing to categorise coding sequences as COX1 marker sequences

*gene* and *gene_synonym* qualifiers			*product* qualifier
Exact match	Prefix match	Pattern match	Exact match
CYTC1	COX1/	(MT[\s]?)?CO[X]?[\s]?[1I]	CYTOCHROME OXIDASE SUBUNIT 1
CYTC-1	COX1&	CYTOCHROME.*OXIDASE SUBUNIT 1	COX1
CYT-C1	COX1_	CYTOCHROME.*OXIDASE SUBUNIT I	COX1 PROTEIN
COX1A	COX1-		COX1P
COX1B	COX1.		CYTC1
			COXI
			COXI PROTEIN

### Community support

ENA operates a highly collaborative model for providing services to specialist user groups. In the context of ENA's advanced search, such collaborations have led to the addition of requested fields from the MIxS and Micro B3 sample checklists, as part of the MG Portal (6) and Micro B3 projects, the non-coding domain and provision of MD5 checksums for samples in output reports, as part of the RNAcentral project (7,8) and the marker portal, which supports the exploration of phylogenetic marker sequences, under the i4Life project (http://www.i4life.eu).

### Data access

**Figure 3.**
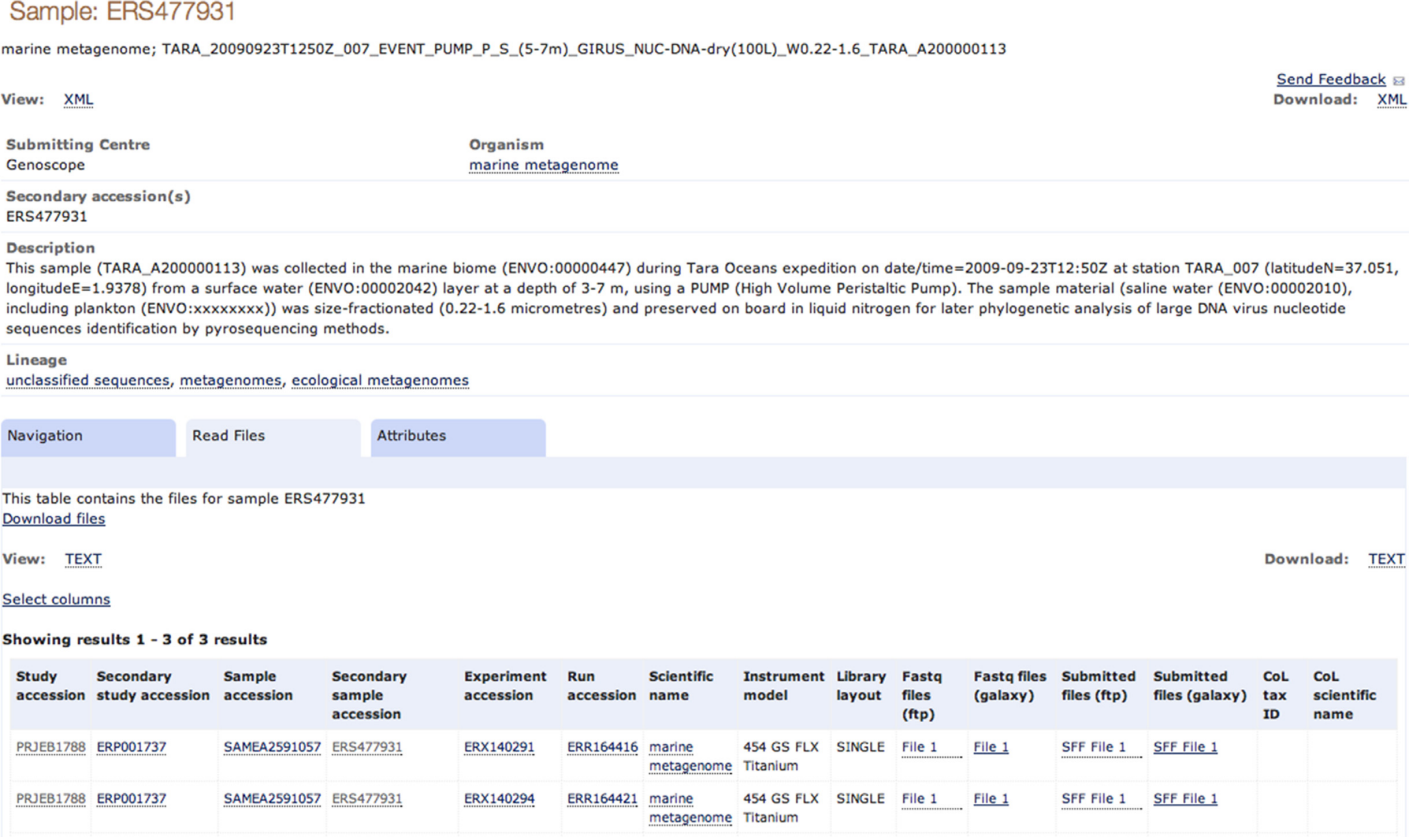
An example sample object in the ENA browser, illustrating integration of data and related content.

ENA content is presented in rich browser, programmatic and file transfer environments, all available from http://www.ebi.ac.uk/ena. We strive for a clear and consistent representation with high levels of integration between records and a variety of download options. Figure 3 shows an example of this integration at the level of a sample record, for which related content such as taxon, parent study and derived data are linked, and provides options to download appropriate information in a variety of formats.
